# Diagnostic Reassessment of a Pulmonary Mass Initially Suspected to Represent IgG4-Related Lung Disease: The Complementary Role of Tissue Metagenomic Sequencing

**DOI:** 10.3390/diagnostics16183018

**Published:** 2026-09-17

**Authors:** So-Yun Kim, Joo-Eun Lee, Da Hye Lee, Hyun-Yi Kim, Kyung-Hee Kim, Hyeongseok Jeong, Chaeuk Chung

**Affiliations:** 1 Division of Pulmonology and Critical Care Medicine, Department of Internal Medicine, College of Medicine, Chungnam National University, Daejeon 35015, Republic of Korea; 2 Department of Biomedical Research Institute, Chungnam National University Hospital, Daejeon 35015, Republic of Korea; 3 NGeneS Inc., Ansan-si 15495, Republic of Korea; 4 Department of Pathology, Translational Immunology Institute, College of Medicine, Chungnam National University, Daejeon 35015, Republic of Korea; 5 Division of Infectious Diseases, Department of Internal Medicine, College of Medicine, Chungnam National University, Daejeon 35015, Republic of Korea

**Keywords:** pulmonary mass, subacute invasive aspergillosis, IgG4-related lung disease, metagenomic next-generation sequencing (mNGS)

## Abstract

**Background/Objectives**: Pulmonary mass-like lesions may mimic malignancy, infection, or inflammatory disease, complicating diagnosis when radiologic, histopathologic, and microbiologic findings overlap. This case illustrates the importance of diagnostic reassessment when the clinical course and treatment response are not fully consistent with the initial diagnosis. **Methods**: A 69-year-old man with diabetes mellitus and mild emphysema presented with chronic cough, purulent sputum, and a recurrent left upper lobe mass. Computed tomography-guided biopsy revealed dense lymphoplasmacytic infiltration with eosinophils, fibrosis, and increased IgG4-positive plasma cells, leading to a presumptive diagnosis of IgG4-related lung disease. Limited improvement with steroid therapy and recurrent hemoptysis prompted multidisciplinary reassessment, including tissue metagenomic next-generation sequencing (mNGS). **Results**: Imaging findings were compatible with subacute invasive aspergillosis, although conventional fungal staining, culture, antigen testing, serologic studies, and bronchial washing analyses were negative. Tissue mNGS detected 348 low-abundance *Aspergillus*-derived reads, accounting for 0.03% of non-host reads. Following initiation of voriconazole, the lesion decreased from 47 mm to 32 mm within six weeks and regressed further thereafter. Symptoms resolved, and hemoptysis did not recur. **Conclusions**: This case highlights the importance of diagnostic reassessment when findings and treatment response do not fully support the initial diagnosis. Tissue mNGS may provide complementary evidence for probable subacute invasive aspergillosis when interpreted alongside radiologic findings, exclusion of alternative diagnoses, and treatment response.

## 1. Introduction

Pulmonary mass-like lesions may represent malignancy, infection, or inflammatory disease, and differentiation can be challenging because radiologic, histopathologic, and microbiological findings may overlap, remain inconclusive, or be discordant [1,2]. Conventional diagnostic approaches, including tissue biopsy [3,4] and microbiological studies [5], may fail to establish a definitive diagnosis, particularly in localized or low-burden infections [6], or after prior treatment [7].

Among infectious causes, pulmonary aspergillosis may present as a mass-like lesion and mimic both lung malignancy and inflammatory lung disease [8,9]. Establishing the diagnosis can be particularly difficult when histopathologic findings are nonspecific and conventional fungal studies, including staining, culture, antigen testing, and polymerase chain reaction, yield negative results.

Advances in molecular diagnostic technologies have provided additional tools for evaluating diagnostically challenging pulmonary lesions. Among these, tissue metagenomic next-generation sequencing (mNGS) may offer complementary microbiological evidence when conventional diagnostic tests are unrevealing [10,11,12]. However, its findings, particularly low-abundance microbial signals, should be interpreted in conjunction with the clinical course, imaging findings, histopathology, and results of conventional microbiological testing.

Herein, we report a case of clinically probable subacute invasive pulmonary aspergillosis (SAIA) initially treated as presumed IgG4-related lung disease, in which recurrent hemoptysis prompted multidisciplinary diagnostic reassessment and tissue mNGS contributed supportive evidence for an *Aspergillus*-related process.

## 2. Case Presentation

A 69-year-old man with hypertension, type 2 diabetes mellitus, and a prior cerebral infarction (on aspirin) presented in November 2024 with chronic cough and purulent sputum. Chest computed tomography (CT) revealed a solid lesion in the left upper lobe (LUL) with mild background emphysema.

**Figure 1:**
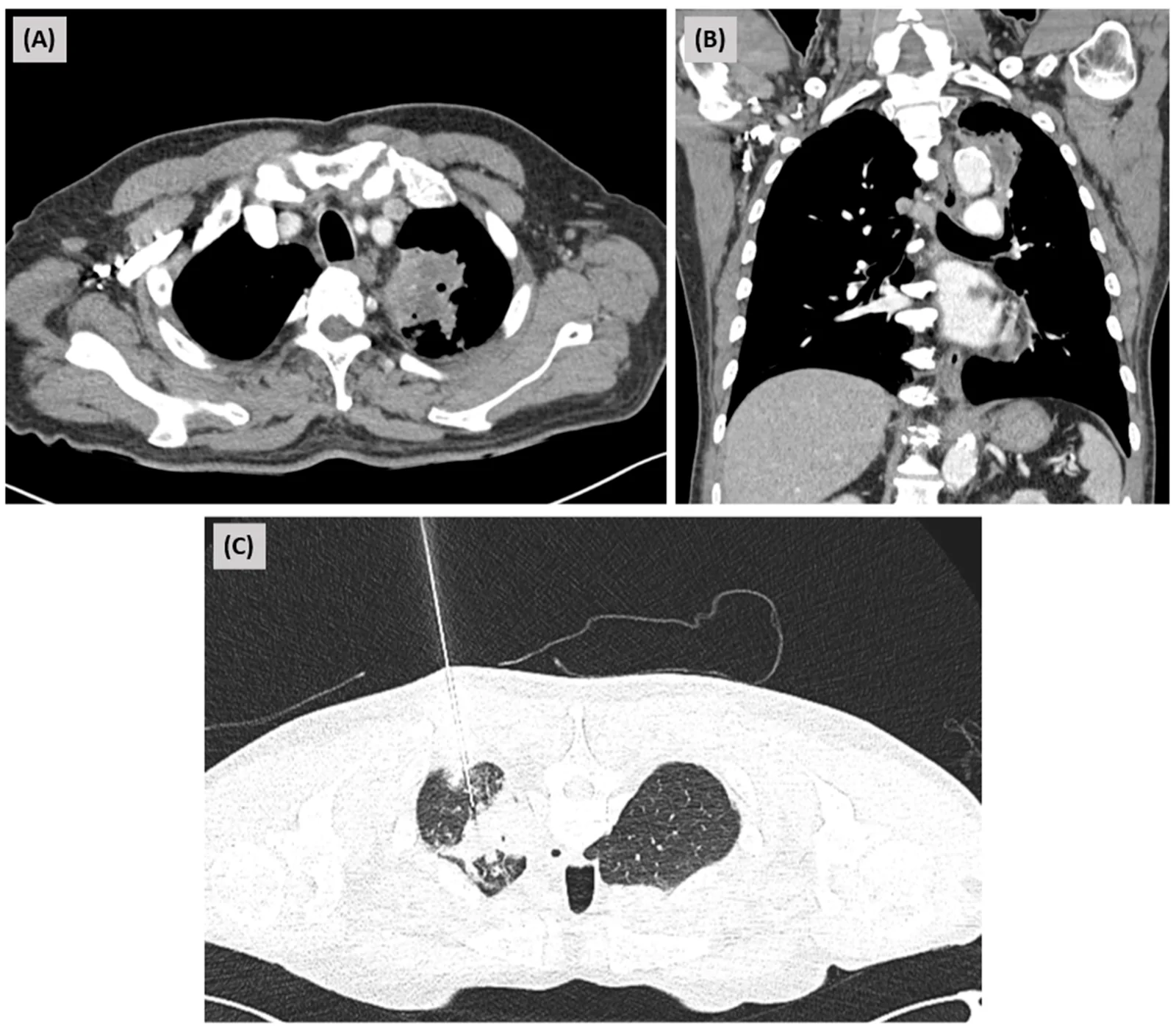
Initial imaging findings and biopsy procedure at an outside hospital. (**A**) Axial contrast-enhanced chest CT image demonstrating a mass-like lesion in the left upper lobe. (**B**) Coronal reconstructed CT image showing the extent of the lesion in the left upper lobe. (**C**) CT-guided percutaneous core needle biopsy performed for tissue diagnosis of the lesion.

Following empirical antibiotic therapy, the lesion partially regressed but subsequently recurred in March 2025 (Figure 1A,B). Because malignancy could not be excluded, a CT-guided percutaneous core needle biopsy was performed at an outside hospital (Figure 1C). Histopathologic examination at the referring hospital demonstrated lymphoplasmacytic infiltration with eosinophils and more than 10 IgG4-positive plasma cells per high-power field, although the IgG4/IgG ratio was below 40%. Based on these findings, IgG4-related lung disease (IgG4-RLD) was initially considered the most likely diagnosis at the referring hospital. The biopsy procedure was complicated by hemoptysis requiring bronchial artery embolization (BAE). Despite two BAE procedures, bleeding persisted, and the patient was referred to our institution for further management.

At our hospital, surgical intervention was considered. However, thoracic surgical evaluation indicated that the lesion was closely adherent to the aorta, making surgical resection high risk. The patient was therefore managed conservatively in the intensive care unit with close monitoring. Given the presumptive diagnosis of IgG4-RLD made at the referring hospital, methylprednisolone at a dose of 1 mg/kg was continued together with empirical antibiotic therapy. Hemostasis was eventually achieved, and the patient was transferred to the general ward.

**Figure 2:**
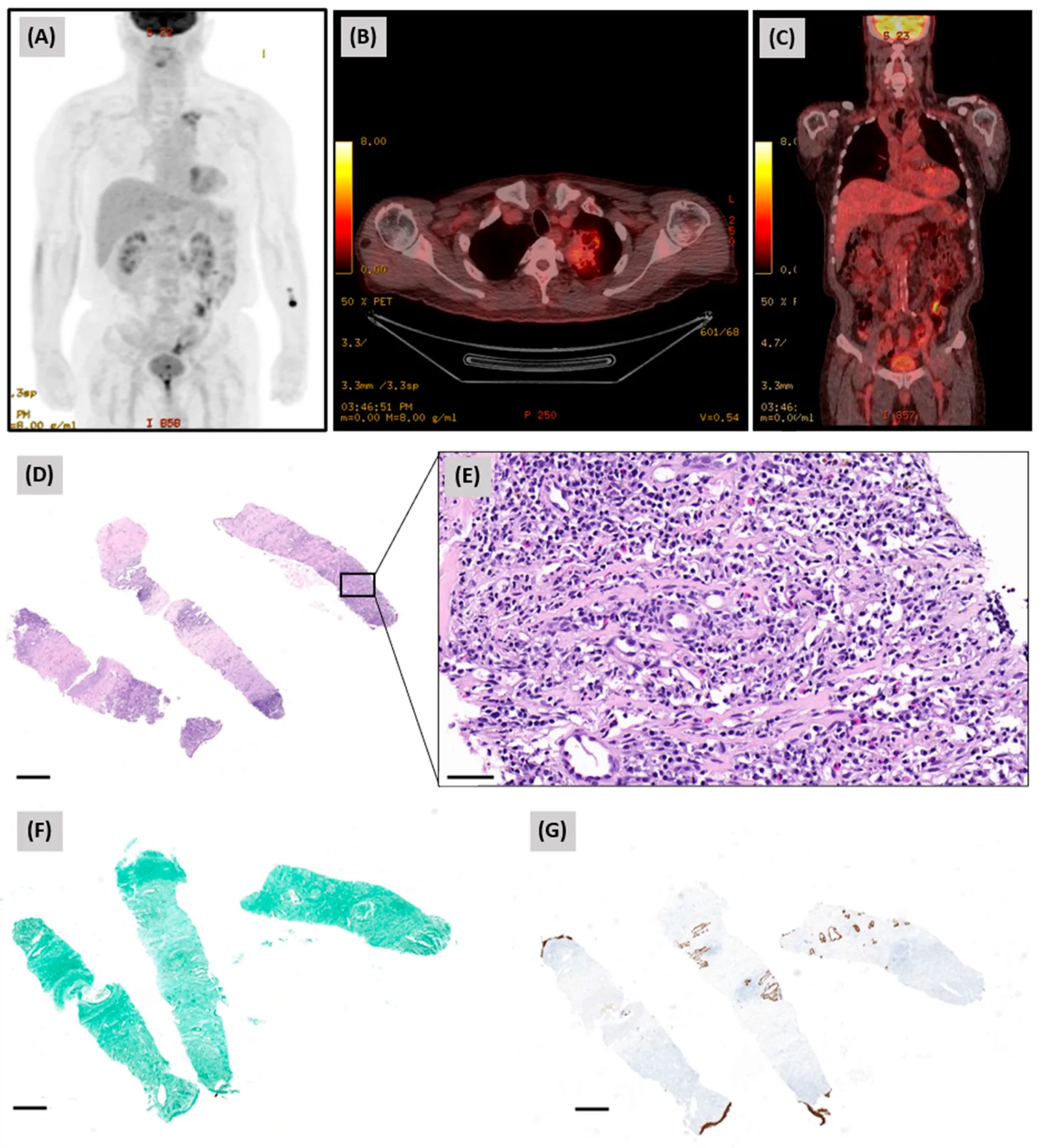
PET-CT findings and histopathologic evaluation of the lung lesion. (**A**) Maximum intensity projection image of PET-CT demonstrating hypermetabolic lesions. (**B**) Axial fused PET-CT image showing increased FDG uptake in the left upper lobe. (**C**) Coronal fused PET-CT image demonstrating no abnormal FDG uptake in the pancreas or biliary tract suggestive of extrapulmonary IgG4-related disease. (**D**) Low-power view of the biopsy specimen (×2) showing fragmented lung tissue with inflammatory changes (scale bar = 500 μm). (**E**) High-power view (×30) revealing dense lymphoplasmacytic infiltration with eosinophils, capillary proliferation, and fibrosis (scale bar = 50 μm). (**F**) Grocott methenamine silver staining showing no fungal elements (scale bar = 500 μm). (**G**) Cytokeratin staining negative for malignancy (scale bar = 500 μm).

Positron emission tomography-computed tomography (PET-CT), performed to further evaluate the possibility of malignancy, demonstrated multiple hypermetabolic lesions in the apical segment of the LUL, raising concern for primary lung cancer (Figure 2A–C).

The referred biopsy specimen was reviewed by pulmonary pathologists at our institution. Histopathologic examination showed dense lymphoplasmacytic infiltration with eosinophils, capillaritis, and fibrosis (Figure 2D,E). Grocott methenamine silver staining revealed no fungal elements (Figure 2F), and cytokeratin immunostaining showed no evidence of malignancy (Figure 2G). On histopathologic review, the findings were nonspecific and insufficient to establish a definitive diagnosis of IgG4-RLD. The previously reported increase in IgG4-positive plasma cells was considered in the overall clinicopathologic assessment; however, given the absence of storiform fibrosis and obliterative phlebitis, the normal serum IgG4 level of 364.8 mg/L (reference range, 39.2–864 mg/L), and an IgG4/IgG ratio below 40%, the overall findings were considered insufficient to establish IgG4-RLD.

Although the diagnosis remained uncertain, the patient showed clinical improvement during steroid therapy and was discharged with a tapering regimen. Follow-up chest CT demonstrated partial regression of the lesion. After stabilization, bronchoscopy with bronchial washing was performed; however, repeat tissue biopsy was deferred because of the high risk of recurrent bleeding. Acid-fast bacilli staining, mycobacterium tuberculosis and nontuberculous mycobacterial polymerase chain reaction testing, direct fungal microscopy, fungal culture, and *Aspergillus* antigen testing of the bronchial washing fluid were all negative. Serum *Aspergillus* antigen, *Aspergillus* antibody, and β-D-glucan tests were also negative. 

Three months later, however, the patient presented again with hemoptysis. Repeat angiography did not identify a definite bleeding focus. Because the symptoms recurred despite steroid therapy, the case was reassessed through multidisciplinary discussion among pulmonologists, radiologists and pathologists. On radiologic review, the lesion showed features compatible with SAIA.

**Figure 3:**
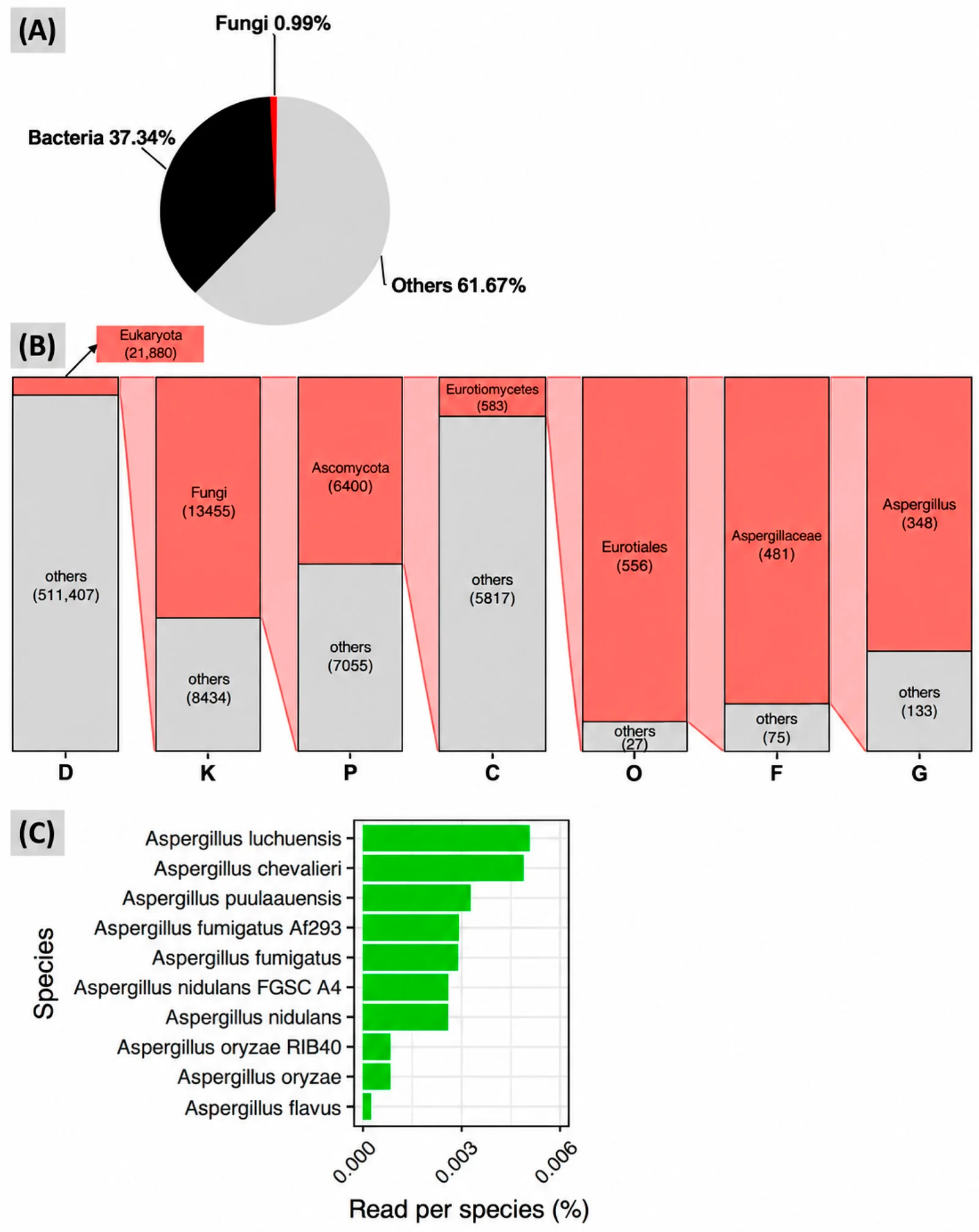
Metagenomic next-generation sequencing (mNGS) analysis of the lung biopsy specimen. (**A**) Distribution of sequencing reads across major microbial categories. Reads were classified using Kraken2 and grouped into user-defined categories (Bacteria, Fungi, and Others) for descriptive analysis; these categories represent different taxonomic ranks and are not directly comparable. (**B**) Taxonomic classification of fungal reads across hierarchical levels (domain to genus), demonstrating the presence of *Aspergillus*-related sequences. (**C**) Relative abundance of detected *Aspergillus* species.

As conventional histopathologic, bronchoscopic, and serologic investigations had failed to provide evidence of aspergillosis, mNGS was performed on the previously obtained biopsy tissue to obtain additional microbiologic evidence. Tissue mNGS, with a turnaround time of approximately 3–4 weeks, detected low-abundance *Aspergillus*-derived sequences, comprising 348 reads and accounting for 0.03% of non-host reads. The reads were distributed across multiple *Aspergillus* species without a single dominant species (Figure 3A–C). Alignment to *Aspergillus fumigatus* showed that 52 of 82 reads (63.4%) mapped to the reference genome across multiple loci on six of eight chromosomes, although the overall breadth of genomic coverage was very low (0.007%) (Supplementary Figure S1). A summary of the diagnostic evaluations and their implications for the differential diagnosis is provided in Table 1.

Given the absence of an alternative diagnosis, the compatible radiologic findings, the recurrent hemoptysis, and the supportive tissue mNGS result, the case was further discussed with infectious disease specialists. Following this multidisciplinary reassessment, empirical oral voriconazole was initiated in July 2025 at a dose of 200 mg twice daily for suspected pulmonary aspergillosis.

**Figure 4:**
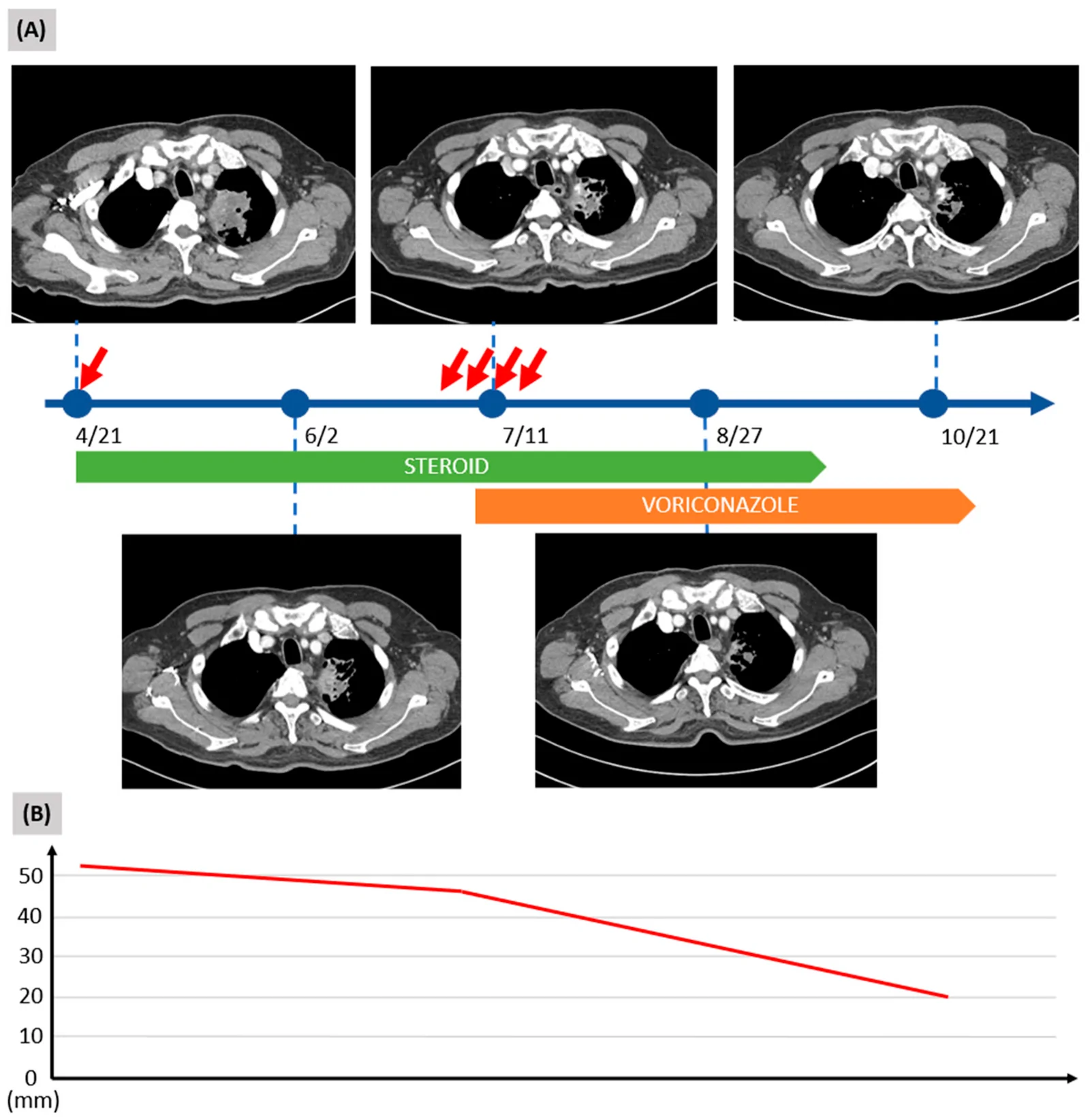
Serial radiologic changes and clinical course of the pulmonary lesion. (**A**) Serial chest CT images showing interval changes in the pulmonary lesion in relation to the clinical course and treatment timeline. Red arrows indicate episodes of hemoptysis. The lesion showed partial interval regression during the period of steroid therapy, followed by further decrease in size after initiation of voriconazole. No recurrent hemoptysis was observed after antifungal treatment. (**B**) Changes in the size of the target lesion over time. The lesion decreased from 51 mm on 21 April to 47 mm on 11 July during steroid therapy and showed a more substantial reduction to 32 mm on 27 August following voriconazole initiation.

**Figure 5:**
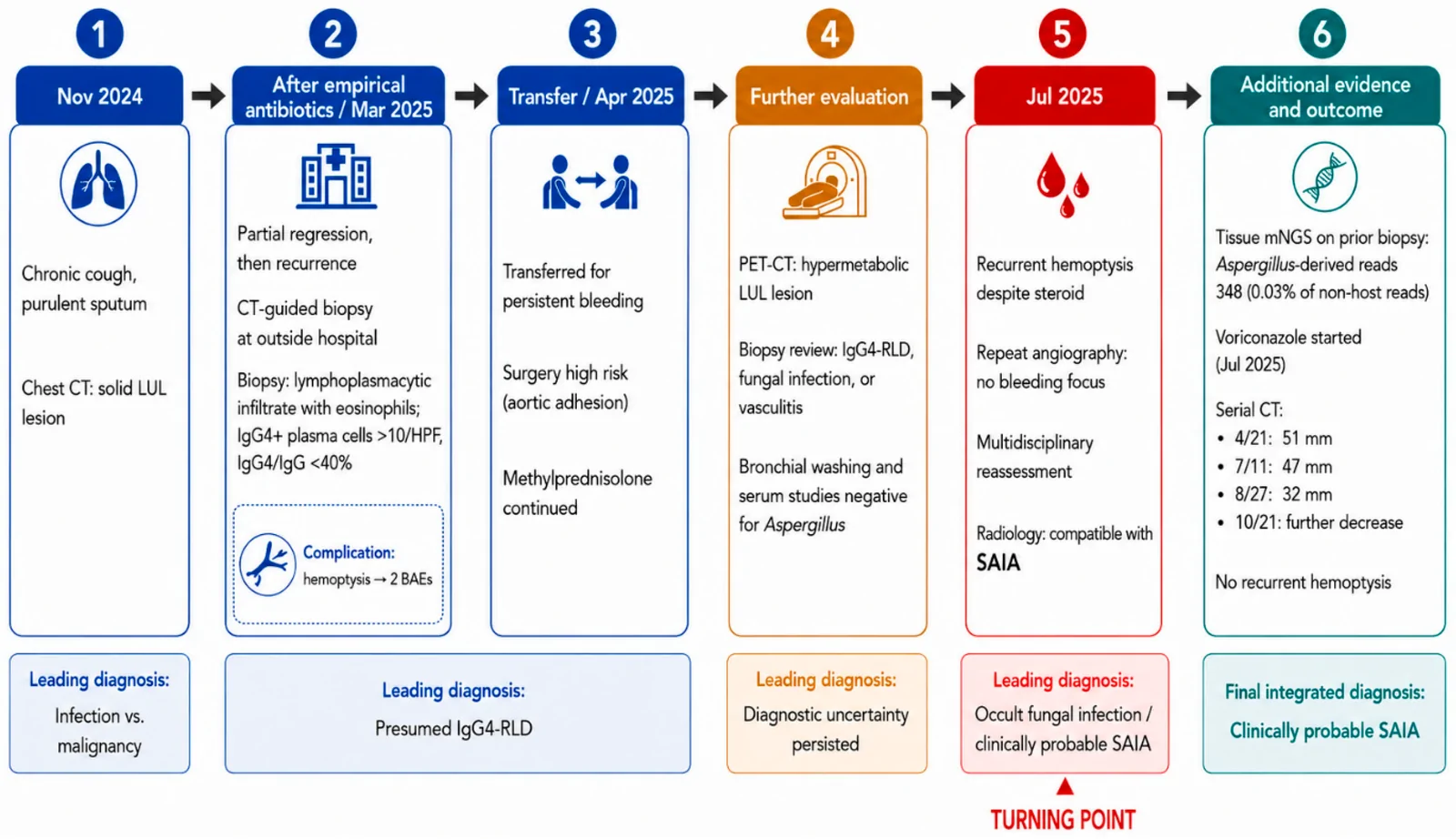
Diagnostic timeline and reassessment process of the pulmonary lesion. The lesion was initially considered to represent either infection or malignancy and subsequently led to a presumptive diagnosis of IgG4-RLD based on biopsy findings. Despite steroid therapy, recurrent hemoptysis prompted multidisciplinary reassessment. Radiologic review raised concern for SAIA, and tissue mNGS provided complementary microbiologic evidence by detecting low-abundance *Aspergillus*-derived reads. The lesion subsequently showed marked regression following voriconazole therapy, supporting a final integrated diagnosis of clinically probable SAIA. This figure prepared with the assistance of ChatGPT (GPT-5.6 Sol; OpenAI, San Francisco, CA, USA)., including generative AI-based image creation, for graphical illustration and layout. All clinical content, diagnostic sequence, and interpretation were determined and verified by the authors. CT, computed tomography; LUL, left upper lobe; BAE, bronchial artery embolization; IgG4-RLD, IgG4-related lung disease; PET-CT, positron emission tomography-computed tomography; SAIA, subacute invasive aspergillosis; mNGS, metagenomic next-generation sequencing.

After voriconazole initiation, the pulmonary lesion showed a more rapid reduction in size than during the preceding steroid treatment period. Serial CT measurements demonstrated only slight regression during steroid therapy, from 51 mm on 21 April to 47 mm on 11 July. After voriconazole initiation, the lesion decreased more substantially to 32 mm on 27 August, when cavitary change became more apparent and an intracavitary nodular component suggestive of fungal material was more clearly visualized. By 21 October, the lesion had further decreased in size. These radiologic changes were accompanied by resolution of symptoms and no recurrent hemoptysis despite discontinuation of steroid therapy. Voriconazole was continued for three months (Figure 4 and Figure 5).

## 3. Discussion

This case illustrates the diagnostic difficulty of a pulmonary mass-like lesion in which malignancy, IgG4-RLD, and fungal infection remained competing considerations. Despite nonspecific histopathologic findings and repeatedly negative conventional fungal studies, recurrent hemoptysis during steroid therapy prompted multidisciplinary reassessment. The combination of radiologic features compatible with SAIA, low-abundance *Aspergillus*-derived sequences detected by tissue mNGS, exclusion of alternative diagnoses, and subsequent clinical and radiologic response to voriconazole supported a diagnosis of clinically probable SAIA.

IgG4-RLD was initially considered because the biopsy specimen showed dense lymphoplasmacytic infiltration with eosinophils, fibrosis, capillaritis, and an increased number of IgG4-positive plasma cells. IgG4-related disease is a systemic fibroinflammatory disorder characterized histopathologically by dense lymphoplasmacytic infiltration, storiform fibrosis, obliterative phlebitis, and increased IgG4-positive plasma cells [13,14]. Pulmonary involvement may manifest as nodules, mass-like lesions, consolidations, interstitial abnormalities, or lymphadenopathy and may therefore mimic malignancy or infection [15,16]. Importantly, increased IgG4-positive plasma cells are not specific for IgG4-related disease and may be encountered as a reactive finding in or adjacent to infectious and other inflammatory lesions [17]. In the present case, the IgG4/IgG ratio was below 40%, the serum IgG4 level was within the normal range, and characteristic histopathologic findings such as storiform fibrosis and obliterative phlebitis were absent. In addition, there was no convincing evidence of extrapulmonary organ involvement, including the pancreas or biliary tract. Taken together, these findings were insufficient to establish IgG4-RLD as the primary diagnosis and underscored the need to interpret IgG4-positive plasma cell infiltration in conjunction with serologic, radiologic, systemic, and histopathologic findings.

The recurrence of hemoptysis despite steroid therapy was a key turning point in the diagnostic process. Although the lesion had shown slight regression during steroid treatment, this response was not disease-specific and did not exclude an underlying infectious process. The recurrence of hemoptysis despite ongoing steroid therapy therefore prompted multidisciplinary reassessment and raised concern for an occult fungal process.

SAIA, historically referred to as semi-invasive or chronic necrotizing pulmonary aspergillosis, is a slowly progressive form of pulmonary aspergillosis characterized by localized tissue invasion over a period of weeks to a few months. It typically occurs in patients with underlying structural lung disease, diabetes mellitus, or mild-to-moderate immunosuppression rather than in those with profound neutropenia [18,19,20]. Radiologic findings may include progressive consolidation, mass-like opacity, cavitation, and pleural or pericavitary inflammatory change. Although histopathologic demonstration of hyphal invasion or microbiologic confirmation can establish the diagnosis, conventional tests may remain negative, particularly in localized disease with a low fungal burden. Serum galactomannan, β-D-glucan, fungal culture, and other conventional assays therefore cannot reliably exclude SAIA when the clinical and radiologic findings are suggestive [21]. In this case, SAIA was considered because the lesion showed a subacute, persistent mass-like consolidative pattern on serial imaging, together with recurrent hemoptysis and supportive tissue mNGS findings. However, the radiologic findings were not specific, and other chronic pulmonary *Aspergillus* syndromes could not be completely excluded on imaging alone.

Tissue mNGS provided complementary microbiologic information but was not interpreted as definitive evidence of infection in isolation. This technique has increasingly been used to identify potential pathogens in culture-negative infections, particularly when conventional diagnostic tests are unrevealing or the microbial burden is low [10,11,12]. Previous reports have similarly demonstrated the potential utility of mNGS in *Aspergillus* infections when conventional microbiologic studies were unrevealing. Huang et al. reported pleural aspergillosis diagnosed using mNGS in a patient with unexplained pleural effusion, while Deng et al. described an *Aspergillus fumigatus* mediastinal abscess in which mNGS contributed to the diagnosis despite diagnostic uncertainty [11,12]. These reports support the potential role of mNGS as a complementary tool in diagnostically challenging or culture-negative *Aspergillus* infections.

In the present case, tissue mNGS detected 348 *Aspergillus*-derived reads, accounting for only 0.03% of non-host reads. Although the signal was of very low abundance, the reads showed taxonomic continuity across the fungal hierarchy, from Fungi through Ascomycota and Eurotiomycetes to the genus *Aspergillus*, suggesting a reproducible genus-level signal rather than reliable species-level identification. Additional reference alignment also showed that 52 of 82 *Aspergillus*-classified reads mapped to multiple loci across six of eight chromosomes of the A. fumigatus reference genome, although overall genomic coverage remained extremely low. These findings provided some additional support for the molecular signal but did not establish invasive infection.

Although detection from biopsy tissue was considered more supportive of a clinically relevant *Aspergillus*-related process than detection from respiratory secretions alone, the absence of histologic fungal invasion meant that colonization could not be definitively excluded. Moreover, because no extraction blank or sequencing negative control was available, environmental or reagent contamination also remained possible. Therefore, the mNGS finding was interpreted only in conjunction with the compatible radiologic findings, recurrent hemoptysis, exclusion of alternative diagnoses, and subsequent response to voriconazole. Accordingly, mNGS was regarded as supportive rather than confirmatory evidence of aspergillosis.

The clinical and radiologic course after voriconazole initiation provided additional support for an *Aspergillus*-related pulmonary process. Although a delayed steroid effect cannot be completely excluded, the lesion showed only modest regression during steroid therapy, decreasing from 51 to 47 mm, whereas a more substantial reduction from 47 to 32 mm occurred within approximately six weeks after voriconazole initiation. Moreover, recurrent hemoptysis ceased despite discontinuation of steroids. Thus, the subsequent clinical and radiologic course was considered supportive of an antifungal response, although it does not establish causality.

This case has several limitations. First, histologic evidence of fungal invasion was not demonstrated, and conventional fungal studies remained negative. Second, the mNGS signal was of very low abundance and no extraction blank or sequencing negative control was available; therefore, contamination, colonization, or bioinformatic misclassification could not be completely excluded. Third, species-level read assignments were obtained, but a specific causative *Aspergillus* species could not be identified with confidence because the reads were distributed across multiple closely related species and genomic coverage was extremely limited. Finally, the favorable response to voriconazole supports, but does not prove, a causal fungal etiology. Accordingly, we use the term “clinically probable SAIA” to reflect an integrated clinical assessment based on radiologic findings, tissue mNGS, exclusion of alternative diagnoses, and longitudinal treatment response, rather than a proven diagnosis established by histopathologic or conventional microbiologic criteria.

## 4. Conclusions

In conclusion, this case highlights the diagnostic challenge of a pulmonary mass that mimicked both malignancy and IgG4-related lung disease despite extensive negative conventional testing. Recurrent hemoptysis during steroid therapy served as an important clinical warning sign and prompted multidisciplinary diagnostic reassessment. Although low-abundance *Aspergillus* signals detected by tissue mNGS should not be regarded as standalone proof of infection, they may provide useful complementary evidence when interpreted together with compatible radiologic findings, exclusion of alternative diagnoses, and the longitudinal clinical response to antifungal therapy.

## Figures and Tables

**Table 1:** Summary of diagnostic evaluations and their implications in the differential diagnosis of the pulmonary mass.

Diagnostic Category	Test or Assessment	Results	Diagnostic Implication
Malignancy	PET-CT	Hypermetabolic uptake	Raised concern for malignancy, but not specific for cancer
	Cytokeratin staining (tissue)	Negative	Malignancy was not supported
	Cytology (bronchoscopy washing)	Negative	
Mycobacterial infection	IGRA (blood)	Negative	Mycobacterial infection was not supported
	AFB stain (bronchoscopy washing)	Negative	
	AFB culture (bronchoscopy washing)	No growth for 6 weeks	
	TB/NTM PCR (bronchoscopy washing)	Negative/Negative	
	TB PCR hybridization (bronchoscopy washing)	Negative	
Autoimmune/connective tissue disease	RA factor (blood)	<2 IU/mL, normal	Autoimmune or connective tissue disease was not supported
	C3 (n) (blood)	107 mg/dL, normal	
	C4 (n) (blood)	33 mg/dL, normal	
	Anti CCP Ab IgG (blood)	8.0 U/mL, negative	
	Anti Jo-1 Ab (blood)	<0.30, negative	
	ANCA (Anti-MPO) (blood)	0.1, negative	
	ANCA (Anti-PR3) (blood)	0.1, negative	
	FANA (blood)	Negative	
IgG4-related disease	IgG4-positive plasma cells (tissue)	>10 per high-power field	Raised concern for IgG4-related disease
	IgG4 (blood)	364.8 mg/L, normal	IgG4-related lung diseaseRLD was not supported.
	IgG4/IgG ratio (tissue)	<40%	
	Storiform fibrosis and obliterative phlebitis (tissue)	Absent	
Fungal infection	*Aspergillus* Ag (blood)	0.12, negative	Conventional fungal studies did not confirm fungal infection
	*Aspergillus* Ab (blood)	27 mg/L, negative	
	Glucan (blood)	<10, negative	
	Fungus direct Microscopy (bronchoscopy washing)	Not found	
	Fungus culture (bronchoscopy washing)	No growth for 3 weeks	
	Pneumocystis jirovecii PCR (bronchoscopy washing)	Negative	
	CT images	possibility of SAIA	Supported clinically probable SAIA when integrated with the clinical course and exclusion of alternative diagnoses.
	mNGS (tissue)	*Aspergillus* reads 348, 0.03% (Low-abundance genus-level signal)	

PET-CT, positron emission tomography-computed tomography; IGRA, interferon-gamma release assay; AFB, acid-fast bacilli; TB, tuberculosis; NTM, nontuberculous mycobacteria; PCR, polymerase chain reaction; RA, rheumatoid arthritis; C3, complement component 3; C4, complement component 4; Anti-CCP, anti–cyclic citrullinated peptide; Anti Jo-1, anti–histidyl-tRNA synthetase antibody; ANCA, antineutrophil cytoplasmic antibody; MPO, myeloperoxidase; PR3, proteinase 3; FANA, fluorescent antinuclear antibody; IgG4, immunoglobulin G subclass 4; CT, computed tomography; SAIA, subacute invasive aspergillosis; mNGS, metagenomic next-generation sequencing.

## Data Availability

All data generated or analyzed during this study are included in this published article.

## Supplementary Materials

The following supporting information can be downloaded at: https://www.mdpi.com/article/10.3390/diagnostics16183018/s1, Figure S1: Distribution of *Aspergillus fumigatus*-aligned reads across the reference genome. Reads taxonomically assigned to the genus *Aspergillus* by Kraken2 were aligned to the A. fumigatus reference genome (GCF_000002655.1). Of 82 reads, 52 (63.4%) were mapped. Blue marks indicate mapped read positions. Despite the very low overall breadth of coverage (0.007%), mapped reads were distributed across multiple distinct loci on six of the eight chromosomes rather than being confined to a single genomic region.

## Abbreviations

mNGS	metagenomic next-generation sequencing
SAIA	subacute invasive pulmonary aspergillosis
IgG4-RLD	IgG4-related lung disease
CT	chest computed tomography
LUL	left upper lobe
PET-CT	Positron emission tomography-computed tomography
