# Supplementary material for: Diagnostic Reassessment of a Pulmonary Mass Initially Suspected to Represent IgG4-Related Lung Disease: The Complementary Role of Tissue Metagenomic Sequencing
Source: Diagnostics (Basel). 2026 Sep 17;16(18):3018. doi: 10.3390/diagnostics16183018 (PMC13605139; doi:10.3390/diagnostics16183018)
Supplement: Supplementary file 1 [file diagnostics-16-03018-s001.zip › diagnostics-4492195-supplementary.pdf]

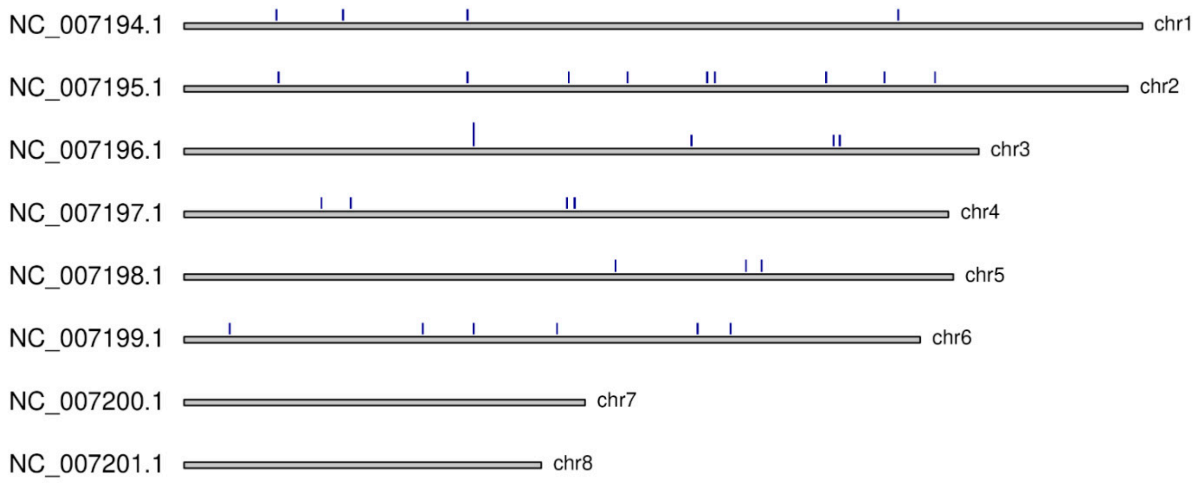

Supplementary Figure S1. Distribution of *Aspergillus fumigatus*-aligned reads across the reference genome.

Reads taxonomically assigned to the genus *Aspergillus* by Kraken2 were aligned to the *A. fumigatus* reference genome (GCF_000002655.1). Of 82 reads, 52 (63.4%) were mapped. Blue marks indicate mapped read positions. Despite the very low overall breadth of coverage (0.007%), mapped reads were distributed across multiple distinct loci on six of the eight chromosomes rather than being confined to a single genomic region.
